# Transcriptomic Changes in Human Tonsil-Derived Mesenchymal Stem Cells Across Culture Passages

**DOI:** 10.3390/genes15121626

**Published:** 2024-12-19

**Authors:** Moon Sik Oh, Heesun Hong, Ok Joo Lee, Su Hyeon Yi, Hae Sang Park, Jae-Jun Lee, Chan Hum Park, Sun-Wha Im

**Affiliations:** 1 Nano-Bio Regenerative Medical Institute, College of Medicine, Hallym University, Chuncheon 24252, Republic of Korea; 2569227@naver.com (M.S.O.); de2509@hallym.ac.kr (H.H.); vudckd@hanmail.net (O.J.L.); hs-piao@hanmail.net (H.S.P.); 2 Department of Biochemistry and Molecular Biology, Kangwon National University School of Medicine, Chuncheon 24341, Republic of Korea; shyun403@gmail.com; 3 Department of Otorhinolaryngology-Head and Neck Surgery, Chuncheon Sacred Heart Hospital, Hallym University College of Medicine, Chuncheon 24253, Republic of Korea; 4 Institute of New Frontier Research, Hallym University College of Medicine, Chuncheon 24252, Republic of Korea; iloveu59@hallym.or.kr; 5 Department of Anesthesiology and Pain Medicine, Hallym University College of Medicine, Chuncheon 24252, Republic of Korea

**Keywords:** tonsil, mesenchymal stem cell, transcriptome, in-vitro culture

## Abstract

Background/Objectives: Tonsil-derived mesenchymal stem cells (TMSCs) are in the limelight in regenerative medicine due to their high proliferation and differentiation potential. It is important to conduct studies to determine the optimal conditions for achieving the maximum yield while maintaining the optimal differentiation capacity of TMSCs. Methods: This study explores the impact of serial subculture on TMSCs by analyzing gene expression at passages 2, 4, 6, and 8. For each culture passage, genes with significant differences in RNA expression from previous passages were selected and their characteristics were observed performing enrichment analysis including KEGG (Kyoto Encyclopedia of Genes and Genomes) and Reactome pathway. Results: At each passage, a “cell cycle” term was ranked high with statistical significance in the KEGG and Reactome pathway. Cell cycle gene expression, including Cyclin-dependent kinases (CDKs) and cyclins, increased until passage 6, then decreased by passage 8. The cell cycle is known to be important not only for proliferation but also for determining whether stem cells maintain pluripotency or differentiate into various lineages. Conclusions: The results suggest that cell cycle gene expression can guide the timing for differentiation induction, with passage 6 potentially being a critical point for initiating differentiation.

## 1. Introduction

Mesenchymal Stem Cells (MSCs) are a versatile cell type found in various tissues, primarily derived from bone marrow and adipose tissue. These cells have self-renewal capacity and can differentiate into various cell lineages through specific induction in vitro, including chondrocytes, adipocytes, and osteocytes. This multipotency underscores the significant potential of MSCs in regenerative medicine and tissue engineering applications. MSCs are concurrently defined by their ability to (a) adhere to plastic surfaces, (b) express specific surface markers, and (c) demonstrate multilineage differentiation potential in vitro.

TMSCs have outstanding advantages over MSCs derived from other sources including bone marrow and adipose tissue in terms of collection and utilization. TSMCs are obtained through tonsillectomy, which removes the problematic tonsils. The completely non-invasive nature of tissue collection eliminates the need for an unnecessary intervention. TMSCs exhibit faster proliferation early in culture and maintain this property over longer passages than bone marrow-derived MSCs (BMSCs) or adipose tissue-derived MSCs (AMSCs) [1,2]. It is known that the proliferation and differentiation capacity of TSMCs is not significantly affected by the age of the donor [3]. The proliferation rate and differentiation potential remain stable even after cryopreservation, enabling successful biobanking for potential clinical applications. In addition, TMSCs have many additional strengths for use in regenerative medicine, such as high yield, low immunogenicity, and the ability to differentiate not only into the mesodermal lineage but also into the endodermal and ectodermal lineages such as hepatocytes and neurons [4].

Sufficient yields facilitate the use of multipotent cells in regenerative medicine. Long subcultures can achieve high yields but may reduce stemness and differentiation potential. Therefore, research is needed to determine when and how maximum yield can be achieved while maintaining optimal differentiation capacity, and for this, the characteristics of TMSCs at each passage must be known.

To gain insight into disease mechanisms, treatment responses, or biological systems, it is useful to detect differences in gene expression in two or more groups during the course of a disease, before and after drug treatment, or during cell differentiation. Researchers can observe the expression of a few genes of interest, but looking at the expression of all genes, if possible, can give them a more holistic understanding of biological differences between groups. When measuring RNA expression levels of samples from two or more groups and calculating the difference in expression levels (e.g., fold change) between the groups for each gene, genes that show statistically significant differences are called differentially expressed genes (DEGs). For example, there are genes that are expressed at higher or lower levels in diseased tissue compared to healthy tissue, and there are genes that are expressed at higher or lower levels in untreated cells compared to treated cells. If the characteristics of the two groups are similar and the number of DEGs is very small, the function of individual genes can be examined through literature or database searches, but if the difference between the groups is large, the number of DEGs can exceed several hundred. In this case, enrichment analysis is used to link the DEGs to specific biological processes, pathways, or phenotypes to see how they function through biological mechanisms. KEGG is a database resource that contains biological pathways including metabolism, genetic information processing, cellular processing, and human disease [5]. Reactome is another pathway database. The Reactome project was founded in 2003 and provides a manually curated and peer-reviewed database by experts in each field [6]. KEGG or Reactome pathway analysis is a bioinformatic enrichment approach used to analyze and interpret data (e.g., DEGs) by mapping them to each pathway database.

Here, we measured gene expression levels at passages 2, 4, 6, and 8 of TSMCs during subculture, obtained DEGs, and performed KEGG and Reactome pathway analysis to investigate the transcriptomic characteristics of TSMCs according to culture passages.

## 2. Materials and Methods

### 2.1. Primary Culture of TMSCs

Tonsils were obtained from pediatric patients who underwent tonsillectomy at Chuncheon Sacred Heart Hospital. Tonsils were washed three times with 1X PBS (phosphate-buffered saline, Corning, NY, USA) containing 1% PS (penicillin-streptomycin, Thermo Fisher, Waltham, MA, USA) in 50 mL conical tubes, cut into small pieces with surgical scissors and incubated with 250 U/mL of collagenase type I (Invitrogen, Waltham, MA, USA) and 20 μg/mL of DNase I (Sigma-Aldrich, St. Louis, MO, USA) in DMEM medium (Dulbecco’s Modified Eagle’s Medium, Corning, NY, USA) for 30 min at 37 °C with shaking at 300 rpm. The tonsil-derived total cells were harvested by filtration through a cell strainer (pore size 100 mm; SPL, Pocheon, Republic of Korea) and washed three times by centrifugation (1300 rpm for 5 min at room temperature). The cell pellet was resuspended twice in DMEM containing 20% FBS (fetal bovine serum) and once in DMEM containing 10% FBS. The cell pellet was suspended in DMEM supplemented with 10% FBS and 1% PS, gently layered onto Ficoll-Paque (Histopaque-1077 g/mL, Sigma-Aldrich, St. Louis, MO, USA), and centrifuged at 2000 rpm for 30 min at 24 °C. The cells were plated at a density of 1 × 10^8^ cells per T-175 tissue culture flask (Corning, NY, USA) in DMEM supplemented with 10% FBS and 1% PS. The plated TMSCs were cultured in DMEM supplemented with 10% FBS and 1% PS with changing half-media every 3–4 days, and subcultured when confluency of TMSCs reached 80%.

### 2.2. Culture and Passaging of TMSCs

TMSCs were cultured in complete growth medium until reaching 70–80% confluence, upon which they were passaged. Cell detachment was achieved using TrypLE™ Express Enzyme (1X) supplemented with phenol red, while minimizing trypsin activation by utilizing DMEM Low Glucose (DMEM(L), Gibco) culture medium containing 10% FBS and 1%PS. Following detachment, cells were pelleted by centrifugation at 2000 rpm for 3 min. Cell counting was performed using the LUNA2 automated cell counter (Logos Biosystems, Anyang, Republic of Korea).

For passaging, cells were seeded at a density of 5 × 10^5^ cells per 100 mm dish. The culture medium was replaced every 2–3 days, and cells were passaged based on cell density. Passage was conducted by detaching cells from the culture surface and replating them at the desired density, ensuring continued growth and expansion of the TMSC population.

### 2.3. Numbers of Cell Population Doubling (NCPD)

To determine the proliferative capability of TMSCs, cells collected at the end of each passage were counted using a trypan blue exclusion method. NCPD was then calculated based on the following equation: “NCPD = 3.33 × log (Nt/Ni)” where Nt and Ni are the cell numbers at a specific time point t and at initial seeding (day 0), respectively.

### 2.4. Flow Cytometry Analysis of TMSCs

TMSCs were harvested at a concentration of 0.5–1 × 10^6^ cells and washed with PBS. Cells were then stained with surface monoclonal antibodies using 100 µL of FACS buffer (Biolegend, San Diego, CA, USA). Then cells were mixed with Stemflow™ human MSC Analysis Kit (BD Biosciences, San Jose, CA, USA) according to the manufacturer’s instructions. The following antibodies were utilized in this study: CD44 PE, CD73 APC, CD90 FITC, CD105 PerCP-Cy5.5, and CD11b/CD19/CD34/CD45/HLA-DR PE. The cell suspension with each antibody was incubated for 30 min at 4 °C in the dark. Following two washes, cells were resuspended in 500 µL of FACS buffer for acquisition on a FACS Canto II System (BD Biosciences, Franklin Lakes, NJ, USA). A total of 10,000 cells were counted, and unstained TMSCs and isotype controls were used as negative controls for each wavelength. Data were analyzed using Cell Quest ProTM, FACS DivaTM version 7.0, and Flowjo version 10.10 (BD Biosciences, Franklin Lakes, NJ, USA). Results were presented as the percentage of labeled cells and fluorescence intensity for each individual monoclonal antibody.

### 2.5. RNA Extraction and Microarray

The entire process of RNA preparation and microarray experiments was performed at Macrogen (Seoul, Republic of Korea). RNA was extracted from TMSCs using the trizol method and its purity and integrity were evaluated with a ND-2000 Spectrophotometer (NanoDrop, Wilmington, DE, USA) and an Agilent 2100 Bioanalyzer (Agilent, Palo Alto, CA, USA). Only RNAs extracted with a total amount of 1 µg or more, concentration of 100 ng/µL or more, purity of 1.7 or more, RNA integrity number (RIN) value of 7 or more, and rRNA ratio of 1.0 or more were used in the microarray experiment, and all samples satisfied these criteria. RNA labeling and hybridization were conducted using the Agilent One-Color Microarray-Based Gene Expression Analysis protocol version 6.5 (Agilent, Palo Alto, CA, USA). The hybridization solution was applied to the gasket slide and assembled onto the Agilent SurePrint Human Gene Expression v3 8 × 60K Microarray Kit (Agilent, Palo Alto, CA, USA). The hybridized array was immediately scanned with an Agilent Microarray Scanner D (Agilent, Palo Alto, CA, USA) The microarray data were processed using Agilent Feature Extraction software version 11.0 (Agilent, Palo Alto, CA, USA). After the experiment, the quality was checked for each probe and statistical analysis was performed on probes that pass the criteria in all samples.

### 2.6. Analysis of RNA Expression

The expression value for each probe was converted to a log scale with a base of 2, and this value was quantile normalized for comparison between samples. In order to observe the difference in expression level of each gene by culture passage, the ratio of gene expression was calculated as a fold change value. When the fold change is a value between 0 and 1, it is expressed as a negative reciprocal number to show a clear difference. Because there was only one sample per passage, the *p*-values of DEGs could not be calculated. Genes with an absolute fold change value of 2 or more were defined as DEGs. Within this paper, the DEGs between the nth passage and the next (n + 2)^th^ passage are named DEGs_pnpn+2_ (DEGs_p2p4_, DEGs_p4p6_, and DEGs_p6p8_).

### 2.7. Functional Enrichment Analysis

KEGG and Reactome pathway analysis were performed to find the biological properties of these DEGs [7]. The fundamental hypothesis of functional enrichment analysis using KEGG or Reactome databases is that relevant pathways can be identified when the proportion of DEGs within a specific pathway exceeds what would be expected by random chance.

Enrichment analysis using KEGG databases was performed using g:Profiler, a web-based tool [8]. When a user inputs a list of genes, the tool identifies statistically significant biological pathways from the KEGG database. The tool also performs multiple test corrections using g:SCS (Set Counts and Sizes) to reduce false positive results [9]. The selected pathway was considered significant when the adjusted *p*-value of g:SCS was less than 0.05.

Enrichment analysis using Reactome databases was performed using a web-based tool provided by the Reactome project [10]. The basic assumptions and usage method are similar to those of g:Profiler, and the multiple test corrections method uses the False Discovery Rate (FDR) estimated using the Benjamini–Hochberg approach [11].) In Reactome analysis, pathways with an FDR less than 0.05 were considered significant.

## 3. Results

### 3.1. Flow Cytometry Analysis Results of TMSCs

**Figure 1:**
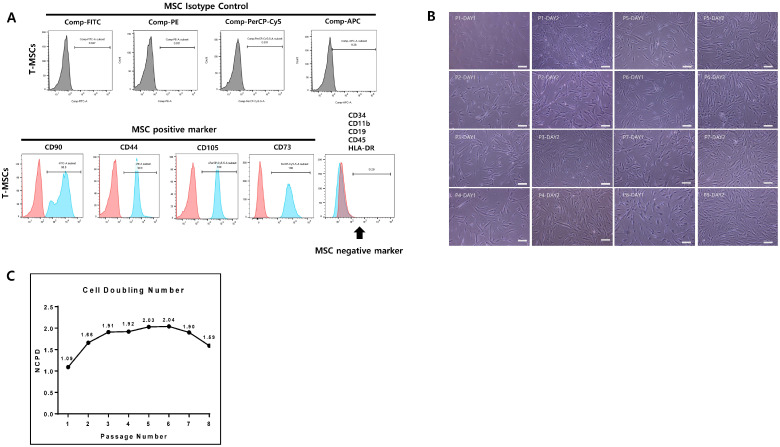
Characterization and proliferative changes of TMSCs. (**A**) Surface marker expression on TMSCs at P4 analyzed using flow cytometry. Red represents the isotype (negative) control, and blue represents the tested sample. (**B**) Representative images of the fibroblast-like morphology of TMSCs at P1–P8 are presented. Scale bar 100 µm. (**C**) Numbers of cell population doubling (NCPD) at each passage.

TMSCs were subjected to flow cytometry analysis according to the described method to identify expression patterns of surface markers (Figure 1A). CD44, CD73, CD90, and CD105 exhibited positive expression, indicating the presence of characteristic MSC markers. Quantitative analysis of the flow cytometry data demonstrated that, on average, 99.9% of cells were positive for CD44, 100% for CD73, 98.8% for CD90, and 100% for CD105. These results confirm the MSC phenotype of the TMSC population. Conversely, negative markers including CD11b, CD19, CD34, CD45, and HLA-DR showed minimal to no expression, consistent with the expected profile for TMSCs. Overall, the flow cytometry analysis corroborated the identity of TMSCs.

### 3.2. Proliferation Rate and Morphology of TMSCs

This study examines the passage-dependent density and morphological alterations of cells cultured for one and two days post-seeding (Figure 1B,C). The proliferation rate showed no overall noticeable change until passage 8; only the NCPD decreased slightly after P6. Similar proliferation patterns were also observed in other studies targeting BMSCs [12,13,14].

Initially, cells exhibited a spindle-shaped morphology resembling fibroblasts. Until passage 6, significant differences in morphology were difficult to discern visually under phase-contrast microscopy. However, from passage 8 onwards, a distinct pattern emerged where cells elongated or became slightly enlarged, adopting irregular and flattened shapes. Moreover, as passage number increased, cells tended to increase in irregularity and hypertrophy, and the nuclei became externally located. Changes in cell morphology during passage progression may reflect changes in cell function and properties. There was a report that inhomogeneous MSCs appeared after passage 6 in BMSC cultures, and another report showed an increase in cell size from passage 7 compared to passage 5 in BMSC [12,14]. These studies targeted BMSCs rather than TMSCs, and the judgment of morphology is somewhat subjective and there is no quantitative index, making direct comparison difficult.

### 3.3. Changes in Gene Expression by Culture Passage

**Figure 2:**
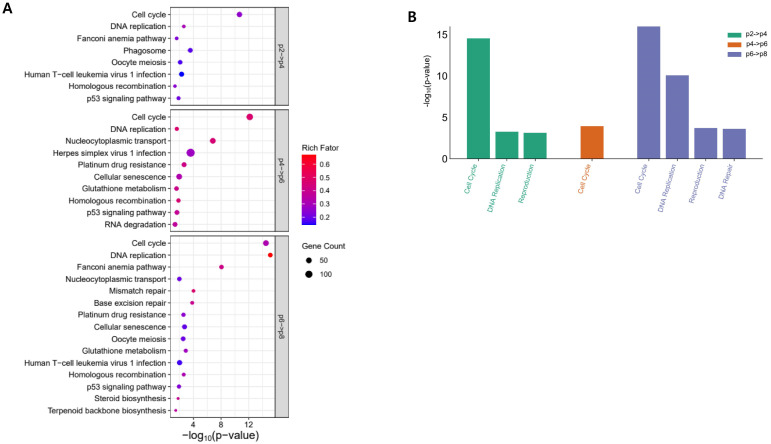
Results of gene enrichment analysis of DEGs. (**A**) Results of KEGG pathway analysis. (**B**) Results of Reactome pathway analysis.

Results of KEGG and Reactome pathway analysis for the DEGs are shown in Figure 2. Of DEGs_p2p4,_ most of the significantly enriched terms of the KEGG pathway were related to cellular processes (cell cycle, phagosome, oocyte meiosis, and p53 signaling pathway) or genetic information processing (DNA replication, Fanconi anemia pathway, and homologous recombination). Genes involved in cellular processes and genetic information processing are pivotal for the proliferation of all cells, including stem cells. DEGs_p4p6_ and DEGs_p6p8_ were also enriched in similar terms of the KEGG pathway to DEGs_p2p4_. Results of Reactome pathway analysis were similar to those of the KEGG pathway (Figure 2B). Cell cycle is consistently enriched at all DEGs. DEGs_p6p8_ were also enriched in DNA repair in the Reactome pathway and mismatch repair and base excision repair in the KEGG pathway.

Additionally, DEGs_p4p6_ and DEGs_p6p8_ were also enriched in other terms related to metabolism in the KEGG pathway, including glutathione metabolism, steroid biosynthesis, and terpenoid backbone biosynthesis.

### 3.4. Cell Cycle Genes

**Figure 3:**
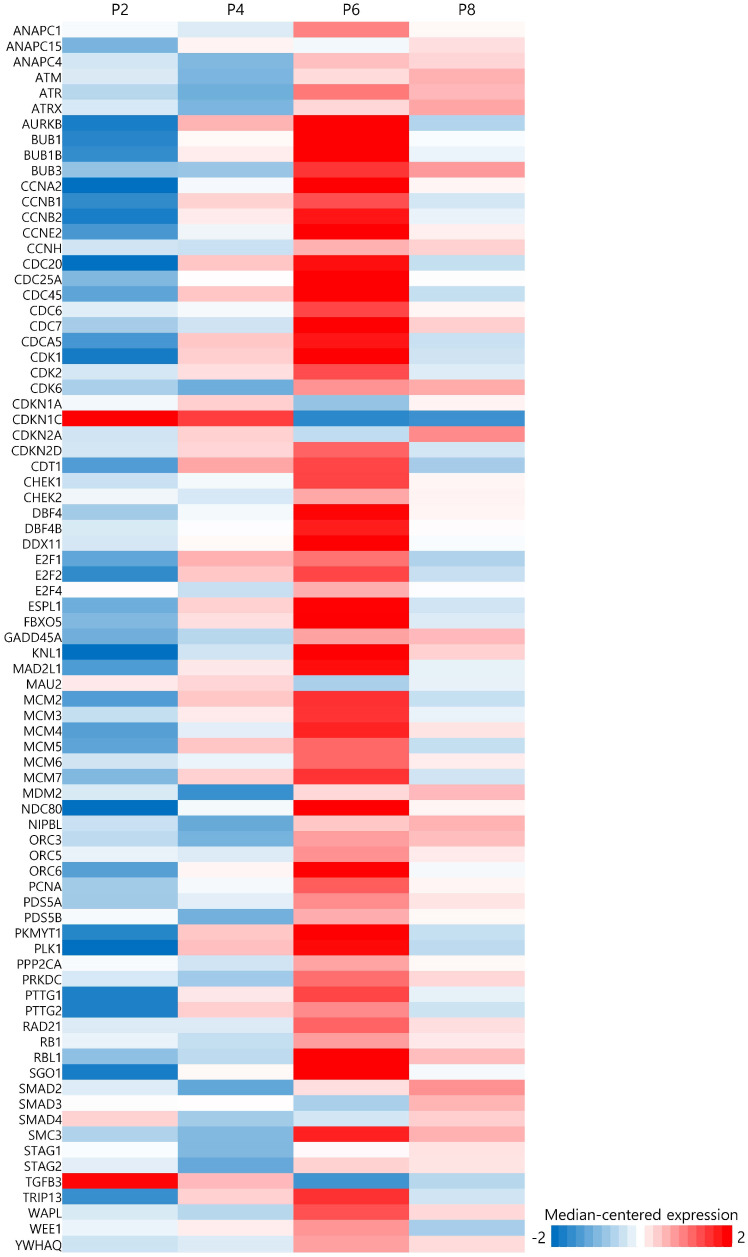
Heatmap of 79 cell cycle genes at each passage.

Changes in expression patterns of cell cycle genes by culture passage are shown in Figure 3. The expression of most genes involved in the cell cycle increased until passage 6 and then decreased at passage 8. However, some genes including *CDKN1C* and *TGFb3* show opposite patterns. There are many kinds of positive and negative cell cycle regulators that promote or inhibit cell division. They are cyclins, cyclin-dependent kinases (CDKs), RB1, E2F, anaphase-promoting complex, INK, and KIP/CIP proteins. Their amount or activity is delicately tuned along the cell cycle, but the pattern seems to be different in somatic cells, early developing embryonic cells, and stem cells [15]. For example, murine or human embryonic stem cells have a shorter G1 phase (3 h) than somatic cells (11 h). Although cyclin E-CDK2 is cyclically expressed and induces somatic cells to enter S phase, murine embryonic stem cells consistently express higher levels of cyclin E than somatic cells throughout the cell cycle. In human embryonic stem cells, cyclin E-CDK2 is highly expressed throughout the G1 and S phases but decreases in the G2 phase. Other cell cycle proteins displayed different patterns in embryonic stem cells from somatic cells.

## 4. Discussion

There have been studies observing transcriptomic or proteomic changes during the serial subculture process of MSCs. When BMSCs were continuously cultured, the expression of genes related to cell cycle, DNA replication, and DNA repair was decreased in late passage (passage 7–11) compared to early passage (passage 2) in all three donors [16]. Another paper comparing the characteristics of passage 4 and passage 10 umbilical cord-derived MSCs also reported changes in the amounts of proteins related to the cell cycle and p53 pathway [17]. In another paper comparing transcriptome differences between control (passage 5–8) and aged (passage 20–25) TMSCs, changes in the expression of genes involved in cell cycle, DNA replication, base excision repair, and the p53 pathway were observed [18]. Although direct comparison is difficult because the sources of MSCs used in these studies and the passages compared were different, it was commonly confirmed that the expression of genes related to the cell cycle, DNA repair, and p53 pathway mainly changed during the serial subculture process of MSCs.

Among the pathways associated with DEGs_p4p6_ and DEGs_p6p8_, there were glutathione metabolism, steroid biosynthesis, and terpenoid backbone biosynthesis. Glutathione is derived from glutamate, cysteine, and glycine, and it functions as a redox buffer that removes toxic peroxides from cells. In a recent study using single cell multiomics data of young and old skeletal muscle stem cells, researchers observed glutathione metabolism perturbed in old skeletal muscle stem cells [19]. Skeletal muscle stem cells consist of both cells with high and low glutathione metabolism. With advancing age, the proportion of cells with low glutathione metabolism increases, and they show declines in both S-phase entry and cell survival, which are rescued by replenishing glutathione. These findings suggest glutathione metabolism as a mechanism regulating stem cell aging and its reversal.

Terpenoids, also called isoprenoids, consist of five carbon isoprene structural units. Terpenoids are precursors of steroids. Steroid hormones bind to nuclear hormone receptors to regulate various physiological processes including energy metabolism, reproduction, and electrolyte homeostasis. Steroid hormones, including estradiol, progesterone, and androgens, work with their receptors to affect stem cell proliferation, differentiation, and survival [20].

It has been suggested that regulation of the cell cycle in stem cells is important not only for rapid division but also for maintaining or inducing a pluripotent state; embryonic stem cells seem to need cyclins A and B for cell division and cyclins D and E for pluripotency. Ablation of cyclin D/E attenuated the pluripotent properties of mouse embryonic stem cells. Cyclin D/E-CDKs phosphorylate and stabilize Oct4, Sox2, and Nanog [21]. When stem cells begin to differentiate into specific lineages, the duration of the cell cycle is prolonged, and the expression of cell cycle genes is generally altered [22]. In the process of somatic reprogramming, in which somatic cells are converted into induced pluripotent stem cells, the cell cycle is accelerated. Ectopic expression of certain cyclins and CDKs or inhibition of RB1, INK, and KIP/CIP proteins promotes somatic reprogramming [23]. Collectively, cell cycle organization appears to be intimately involved or causally involved in maintaining pluripotency or differentiation of stem cells, but the precise mechanisms remain unclear.

There are some limitations in interpreting our results. The experiments in this study using microarray measure only the expression level of individual genes. RNA is translated to make proteins, and proteins go through post-translational modifications such as phosphorylation, glycosylation, acetylation, and lipidation to finally have a function. These proteins act as transcriptional factors to change the expression of other genes or act as enzymes to change the activity of other proteins. For example, regulation of cell cycle genes is achieved not only by expression level but also by activation control mechanisms such as phosphorylation. In this regard, our study results contain fundamental limitations in interpretation. Measuring gene expression through RNA provides important clues to cell state assessment, but there is a limit to estimating the type, amount, and activity of proteins, or the aspect of end products such as steroid hormones that occur after transcription. Therefore, a further multiomics approach that evaluates various aspects of the cells, such as DNA, protein, and lipid, in addition to RNA, will be useful for evaluating the state of the cells.

Even within cells of the same passage, cells of different ages are mixed. When subculture is repeated, fast differentiating cells are decreased and slow differentiating cells are increased. Cells in these different stages also have different patterns of gene expression. When these cells are collected and analyzed together, we observe the combined results of the signal from all these cells. Single cell transcriptome analysis is one way to overcome these limitations. Knowing the differentiation stage of each cell within the stem cell compartment and the fraction of cells that retain pluripotency can provide an important criterion for determining at which passage TMSCs are differentiated into a specific lineage. Ensuring the highest yield in this way can satisfy a major concern of regenerative medicine.

## 5. Conclusions

This study underscores the importance of cell cycle genes in the context of TMSC subculture. By focusing on these genes, researchers and clinicians can better determine the optimal passage for transitioning TMSCs from proliferation to differentiation. This approach can enhance the efficacy of stem cell-based therapies by ensuring that cells are at an appropriate stage for their intended regenerative functions.

## Data Availability

Data will be made available on request.
